# Representing Normal and Abnormal Physiology as Routes of Flow in ApiNATOMY

**DOI:** 10.3389/fphys.2022.795303

**Published:** 2022-04-25

**Authors:** Bernard de Bono, Tom Gillespie, Monique C. Surles-Zeigler, Natallia Kokash, Jeff S. Grethe, Maryann Martone

**Affiliations:** ^1^Auckland Bioengineering Institute, University of Auckland, Auckland, New Zealand; ^2^Department of Neuroscience, University of California, San Diego, San Diego, CA, United States; ^3^Faculty of Humanities, University of Amsterdam, Amsterdam, Netherlands

**Keywords:** anatomy, physiology, pathology, knowledge management, knowledge representation, ontologies, visualization

## Abstract

We present (i) the ApiNATOMY workflow to build knowledge models of biological connectivity, as well as (ii) the ApiNATOMY TOO map, a topological scaffold to organize and visually inspect these connectivity models in the context of a canonical architecture of body compartments. In this work, we outline the implementation of ApiNATOMY’s knowledge representation in the context of a large-scale effort, SPARC, to map the autonomic nervous system. Within SPARC, the ApiNATOMY modeling effort has generated the SCKAN knowledge graph that combines connectivity models and TOO map. This knowledge graph models flow routes for a number of normal and disease scenarios in physiology. Calculations over SCKAN to infer routes are being leveraged to classify, navigate and search for semantically-linked metadata of multimodal experimental datasets for a number of cross-scale, cross-disciplinary projects.

## Introduction

ApiNATOMY is a method that represents biological flow processes as graph edges, and biophysical structures that convey such processes as models of material conduits. Circuit assemblies of ApiNATOMY conduits, and the processes that these conduits convey, are semantically annotated with ontological terms to create FAIR (Wilkinson et al., 2016) topological models of flow from scenarios in normal physiology and disease (e.g., the regulation of peristalsis in the colon, the control of continence of the bladder). In this paper, we give an account of the workflow of ApiNATOMY modeling and some of its applications.

The evolution of the ApiNATOMY method is documented in this tabulation of nine milestones (Table 1). The ApiNATOMY approach of leveraging flow to represent, and to a degree account for, the biological architecture that conveys it originates with the identification of the functional tissue unit (FTU) (de Bono et al., 2013). The FTU was initially determined as a cylindrical tissue parcelation centerd around a blood capillary in which any two points are within diffusion distance. Organizing FTU knowledge over treemaps (de Bono et al., 2012; Kokash et al., 2012), which are directly generated from partonomy and subsumption networks drawn from anatomy ontologies, was a first approach to model in ApiNATOMY whole-body flow-routes inspired by well-established physiology-based pharmacokinetic (PB-PK) methods (e.g., Sager et al., 2015; Jamei, 2016; Thomas et al., 2016).

**TABLE 1:** Timeline for milestones in ApiNATOMY development.

No.	Theme (References)	Description of Milestone(s)
1	Treemaps (de Bono et al., 2012; Kokash et al., 2012)	- Organizing the visual inspection of anatomy ontologies as tessellations to create visually-immediate and anatomically-meaningful treemap layouts representing regions of the mammalian body.
2	Primary FTUs (de Bono et al., 2013)	- Describing, *for the first time, the precise biophysical and topological characteristics of functional units of tissue. Such a unit consists of a three-dimensional block of cells centred around a capillary, such that each cell in this block is within diffusion distance from any other cell in the same block. [.] This block of tissue has a cylindrical shape whose long axis is that of the feeding capillary on which it is metabolically dependent.*
3	Connectivity Routes (Grenon and de Bono, 2011, 2013)	- *We use: i) formalised anatomical knowledge of connectivity between body structures and ii) a formal theory of physiological transport between fluid compartments in order to define and make explicit the routes followed by proteins to a site of interaction.* - *The motivation for the approach can be understood as using knowledge representation and reasoning methods to propose concrete candidate routes corresponding to correlations between variables in mathematical models of physiology.*
4	Visualising Routes As Circuits (de Bono et al., 2014)	*Our approach allows for the consistent diagrammatic rendering of circuitry data, regardless of the scale, modality or species of acquisition. Through ApiNATOMY, the basic circuits utilized by the nervous system to mediate behavior can be revealed, compared and linked to actual data.*
5	As Gatekeepers For Molecular Interaction, FTUs Assemblies Are Fundamental To Study Interactions In Physiology (Hunter and de Bono, 2014)	- *Of the 17 top-level terms in the GO Molecular Function hierarchy, 16 represent functions in which a protein carrying out such a function must effect a change in molecular structure over the course of direct physical interaction with other molecules.* - *Molecular transport and communication between distributed or contiguous primary FTUs via the endothelial or epithelial vessels is characterised at the level of secondary functional tissue units (sFTUs). sFTUs represent units of physiological function that are replicated multiple times in a whole organ.*
6	An Infrastructure For The Semantic Modeling Routes of Flow Physiology (de Bono et al., 2015)	- *The Open Physiology workflow that overcomes* [.] *two formidable representational challenges of tissue structure and function, namely, how to: 1) apportion tissue space in terms of diffusive parcellations* [.], *as well as 2) connect these parcellations across distances that are well beyond the diffusion limit* [.] *to enable modeling of fluid flow that conveys molecules from one organ to another, or across distant tissue regions within the same organ.*
7	Representing Processes In Pathophysiology Through Conduits (de Bono et al., 2016a)	- *Formal representation [.] of the pathophysiology mechanism [.] describes a number of correlations of rate and state measurements drawn from a range of locations along and across [.] conduit systems. The basic organizational features that need to be taken into account to represent routes linking measurement locations are that: (i) key biophysical interactions regulating the constitution, as well as the flow, of fluid in the lumen take place between the Wall (W) of the conduit and the Content (C) contained by the same conduit—two basic types of location, therefore, need to be distinguished: (C, W); (ii) transfers between conduit systems transit through the connective tissue ‘glue’ that organizes bundles of endothelial, epithelial and neural conduits; (iii) the representation of long-range material transfers (i.e. beyond the range of the diffusion limit) must take into account the topology of conduit arborizations to explicitly link tube types from different material properties that communicate along the same conduit system.*
8	Bond Graph Formalism Constraining Flow Models In ApiNATOMY (de Bono et al., 2018)	- *A framework for developing multiscale biophysical models in physiology by combining (i) ApiNATOMY, a representation of functional anatomy, with (ii) bond graphs, an engineering methodology that represents mass and energy-conserving processes consistently and powerfully. We also demonstrate the application of bond graphs in ApiNATOMY by generating and linking three biophysical scenarios from different scales, namely: A. the fluid mechanics of blood and urine flow in the kidney; B. the biochemical and diffusive processes between blood and urine of the proximal tubule of the nephron and C. the multistate dynamics of a membrane transporter in the renal tubular epithelium.*
9	ApiNATOMY Knowledge Representation And Its Implementation (Kokash and de Bono, 2021)	- Presentation of: *[i] the ApiNATOMY conceptual schema; [ii] a model transformation steps to obtain fully instantiated entity-relationship object models from the initial template-based specifications; and [iii] model display.*

*Text in italics is a direct quote from publications referenced in the second column.*

Subsequent refinements to the above Knowledge Representation (KR) in ApiNATOMY included:

• generalizing the definition of an FTU to encompass any 80μm-diameter, 60μm-long cylindrical tissue parcel that longitudinally cuffs long-range exchange vessels consisting of either endothelium, mesothelium, surface epithelium or neuron (Hunter and de Bono, 2014);
• recognizing the generic FTU as a special case of a generic conduit that was instantiated at a particular scale;

extending the definition of conduit to any topologically-cylindrical biological structure (immaterial of scale) that conveys fluid flow (i.e., the subcellular sodium pump, as well as an unbranched segment of the thoracic aorta, are both considered as conduits in ApiNATOMY) (de Bono et al., 2015, 2016a);

• developing a construction kit (Kokash and de Bono, 2021) to build conduit models of complex, branched biological structures in which:
  • conduits are typed to either of three configurations: Tube, Bag or Cyst,
  • representations of vessel arborizations (e.g., urinary tree) are created from linear/serial assemblies of conduits, as well as
  • radial coalescence of conduits to depict shared outer layers between conduits (e.g., to model the interaction between the surface epithelial renal glomerulus and the endothelial Bowman’s capsule).
• recognizing that, while representing the multi-scale flow of fluid material serves as the main PB-PK use case, leveraging the Bond Graph formalism (de Bono et al., 2018) to model flow within ApiNATOMY would extend the representation to include other kinds of biophysical transactions (e.g., enzymatic/electrochemical transforms, or the linear conduction of tensile or compressive forces).

**FIGURE 1:**
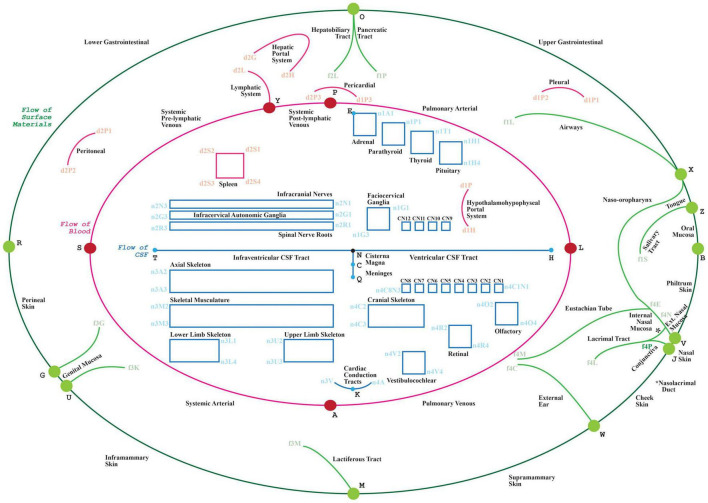
The visual depiction of a TOO map. Lines represent the main thoroughfares of material transit as follows: Green: surface material; Red: blood, lymph and mesothelial fluids; Blue: cerebrospinal fluid; cytosol of cardiac conduction syncytium. Rectangles act as bounding boxes for conduits that belong to anatomical structures as labeled.

In this work, we present the tenth milestone in the development of the ApiNATOMY method: establishing a community-driven production workflow of FAIR, quality-checked connectivity models, and the anchoring of these models onto a reference topological scaffold representing major body-level flow thoroughfares (known as the TOO map, Figure 1). In relation to this workflow:

• the Infrastructure section describes the infrastructure requisite to producing ApiNATOMY connectivity and TOO maps;
• the first part of the Methods (section 3.1) exemplifies its application in building one canonical connectivity model and a canonical TOO map;
• the second part of the Methods (section 3.2) outlines the annotation of the TOO map with disease mechanism terms defined as altered flow between body compartments, as originally envisioned in our earlier work (de Bono et al., 2016a);
• the section 4 outlines the accessing and operation of the resultant SCKAN knowledge graph;
• the application of, and future work on, SCKAN in biomedical knowledge management is addressed in section 5.

## ApiNatomy Infrastructure

### Architecture Overview

#### Operational Setting of the Infrastructure in SPARC

The NIH Common Fund’s effort, Stimulating Peripheral Activity to Relieve Conditions (SPARC) (SPARC, 2014), is targeting medical conditions through the development and application of electroceuticals: electronic devices that stimulate peripheral nerves to rectify diseased organ function (Osanlouy et al., 2021). To this end, SPARC has set out to build ApiNATOMY connectivity maps representing routes relevant to central autonomic control of the viscera, and to semantically imbue these maps with annotations that describe organ structure, function and disease involvement.

These connectivity maps are generated through (i) consultation with SPARC experts to carry out a supervised literature review of the neural circuit system under study and (ii) the leveraging of data acquired by a consortium of SPARC laboratories via different experimental modalities (e.g., tracing studies) and organism species. In relation to SPARC experimental data, ApiNATOMY connectivity knowledge serves to organize and classify metadata about data provenance and experimental conditions.

Given the microscopic, branched and sparse nature of neurons, it is crucial to consider that SPARC experimental procedures, such as the neural tracing studies mentioned above, generate statistical data with inherent limitations about the accuracy of the elicited anatomical location and functional features of neural communication. Consultation with SPARC experts is therefore key to infer approximate topologies of neural routes, given that these approximations put important constraints on ApiNATOMY modeling (see connectivity model example in section 3).

The Common Fund Data Ecosystem (2019), of which SPARC is part, provides a broader operational requirement for SPARC’s connectivity knowledge to help classify and manage a broad range of Common Fund-generated biomedical data including clinical records, laboratory assays, and imaging. A key knowledge management use case for the CFDE is to support discovery of mechanisms in molecular pathology (e.g., cohorts studies that compare sedentary *vs* active life styles) and pharmacology (e.g., teratogenicity) through enhanced FAIRness of heterogeneous biomedical data sets.

#### Representational Setting in SPARC

Knowledge representation in ApiNATOMY is already well-described in Kokash and de Bono (2021). Here we give a brief account of the application of three core ApiNATOMY entity classes in the context of the SPARC effort:

• Flow edge class
  • *Advective flow*: Primarily, SPARC maps depict the regulation of advective flow of materials along, and between, three main types of tissue, namely those tissues that are:
    (1) being monitored (or sensed) by the peripheral nervous system (e.g., mucosa of the small intestine);
    (2) in the central nervous system, integrating and processing afferent information to generate an efferent response (e.g., nuclei in the medulla oblongata);
    (3) reacting to efferent stimuli through contraction (e.g., smooth muscle), endocrine (e.g. adrenaline) or exocrine (e.g., pancreatic juice) secretion, or immunomodulation (e.g., splenic macrophages).
  • *Diffusive flow*: In addition to advective flow, the depiction of diffusive flow of solutes across membranes, or within FTUs, is required to represent mechanisms relevant to action potential signaling, paracrine messaging, and transfers across synapses.

• Conduit class
  • Biological structures (e.g., tissue parcellations) represented in SPARC connectivity maps are depicted as either:
    (1) *Unlayered conduit*: if, say, a tissue parcelation is represented as a homogeneous (and therefore unlayered) blob, then it is considered solely as a conduit of its own intrinsic fluid (i.e., conveying either tissue fluid, cerebrospinal fluid, endoneurial fluid, etc.);
    (2) *Multi-layered conduit*: such a conduit consists of (a) an inner-most layer of transported material that is subjected to the principal flow process which the conduit as a whole is modeled to convey (e.g., blood flow conveyed by a blood vessel, urine flow conveyed by a ureter, cytosol flow conveyed by a neuron) and (b) one or more outer layers representing the conduit wall. In a layered conduit, each individual layer is a route for the transport of its own intrinsic fluid such that:
    (a) the innermost layer is a route for the principal material flow;
    (b) layers of the wall conduct fluids that constitute their material, such as tissue fluid (e.g., in the tunica media layer in an arterial wall) or protein and phospholipid constituents (e.g., in the case of cell membranes).

• Group class
  • Groups are arbitrary collections of conduits and associated flow edges. They allow for the construction and semantic tagging of arbitrary subsets within an ApiNATOMY model (e.g., a neuron).
  • Fundamentally, an individual ApiNATOMY model is a group with some extra information, such as authoring metadata attached.

#### Production Setting in SPARC

To date, the SPARC effort has generated peer-reviewed connectivity maps detailing, for instance, autonomic control of the mammalian stomach, spleen, pancreas, lungs, heart, small intestine, colon, urinary bladder, as well as the sympathetic connectome of the superior cervical ganglion (Bernard de Bono et al., 2021). Ensuring the sustainable management, interpretation, comparison and integration of these independently-generated maps relies on two key strategies:

• the coherent annotation of map conduits with terms from standard reference ontologies for biological structure, and
• the graphical layout of these connectivity models over a communal visual scaffold, the TOO map (Figure 1.) as this scaffold apportions the main advective compartmental thoroughfares in the mammalian body according to the primary material types being transported.

The workflow to author TOO maps and connectivity maps leverages tools and resources detailed in the Infrastructure section below.

### Infrastructure Overview

The overview of conceptual modeling and technological stack to enable support for the development of multiscale connectivity models of physiology is outlined in Figure 2. In the context of anatomy and knowledge representation, this figure shows the transition from global textbook-style image (Figure 2(i)A) to local representation of constituent parts (Figures 2(i)C,D): i.e., from illustrative, conceptual wiring diagrams to formal abstracted connectivity maps and detailed representation of flow edges and conduits. We also want to emphasize here the aspects related to the transition from the conceptual level of anatomy to a practical, FAIR representation of extensive connectivity knowledge in the digital form.

**FIGURE 2(i):**
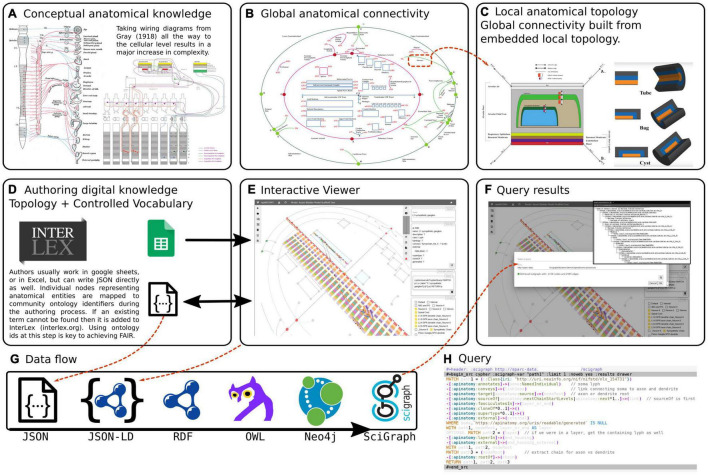
An overview of the ApiNATOMY infrastructure. The top row of three panels illustrates the transition from conceptual to formal representation of physiology knowledge. **(A)** An example of a traditional connectivity wiring diagram. **(B)** Display of TOO Map global scaffold. **(C)** An example of conduit representation in ApiNATOMY. The second row of three panels explains the technical model transformation pipeline. **(D)** The ApiNATOMY authoring process. **(E)** The visualization of an ApiNATOMY model in the graphical viewer after its conversion to the expanded JSON format. **(F)** The results of competency query execution from the visual editor. **(G)** The JSON-to-SciGraph conversion steps. **(H)** Key components of the ApiNATOMY architecture diagram.

**FIGURE 2(ii):**
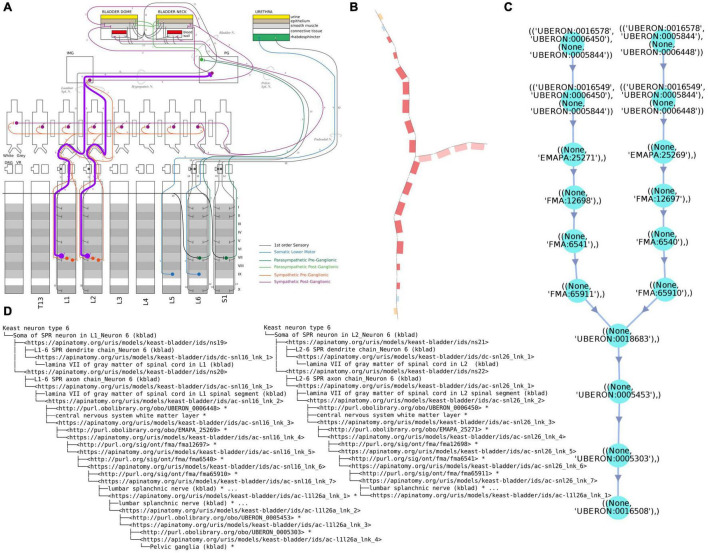
Examples of different renderings of a typical ApiNATOMY query output, in this case for the parts of a neuron population and their locations. All the queries employed here are maintained in Gillespie (2021h). **(A)** “Keast” neuron type 6 (NTK6) highlighted on the original hand drawn diagram. **(B)** NTK6 rendered in the ApiNATOMY viewer. **(C)** SciGraph query results (raw format is JSON, see Supplementary Material) for NTK6 condensed and rendered via CytoScape. **(D)** SciGraph query results for NTK6 rendered as a tree.

Inspired by the graphical diagrams in anatomy textbooks, as well as SPARC experts, we attempt to capture the essential knowledge about multiscale connectivity and render it accessible for computers, findable by other users, and reusable for teaching and research. The connectivity knowledge, initially captured in ApiNATOMY, becomes accessible for reuse and, ultimately, augments many other types of knowledge collected by researchers and practitioners, with the help of our KR transformation JSON-to-Scigraph pipeline (Figure 2(i)G). The key steps in the JSON-to-Scigraph pipeline^1^ are:

• At the very first step, modelers create an intermediate representation (IR) of connectivity data using the conceptual ApiNATOMY JSON Schema, either in JSON directly or in JSON derived from Excel format, described in detail in Kokash and de Bono (2021). The input ApiNATOMY model may include one or two parts: the main part, the *connectivity model*, focuses on the accurate representation of connectivity (Figure 2(i)E). The auxiliary part, *the scaffolding model*, e.g., TOO Map (Figure 2(i)B), provides guidelines on how to display connectivity models to help users to position it into a global context of whole-body topological anatomy.
• Since the explicit, manual definition of all combinations of elements in a connectivity model is unrealistic due to the large number of elements that would have to be individually defined, one of the key features of ApiNATOMY model authoring is the extensive use of *group templates*. These templates are patterns for generating subgraphs, most commonly subgraphs known as *chains* or *trees*. Templates make it possible for authors to work at a level where the concepts they are dealing with are closer to the biological entities that they are trying to represent (e.g., neurons, urinary tree) rather than deal with a sort of anatomical assembly language where biological meaning may be obscured by the abstractions of the ApiNATOMY formalism. Consequently, the second step in our pipeline is to augment the input model by auto-generating implicitly-required resources and relationships. This step often involves massive replication of resources defining conduits and flow edges from predefined templates, and assembling them into a coherent model that accurately portrays its biological counterpart. The result of this step is the representation of an *expanded* model in JSON-LD format. We produce two versions of this model:
  • The first version is a *structured* model that preserves the organization of connectivity knowledge as defined by the user. This model is a blueprint of the connectivity knowledge representation suitable for graphical visualization and interactive manipulation.
  • The second version is the so-called *flattened* model that provides a dictionary of all computational resources that compose the connectivity diagram, with their bidirectional relationships preserved. This model is more convenient for search and interoperability with other knowledge management systems.
• The flattened ApiNATOMY JSON-LD model is directly mapped into RDF/OWL formats and integrated with (i) the Triplestore/Blazegraph database (an ultra high-performance graph database providing RDF/SPARQL APIs) and (ii) SciGraph (the Neo4j graph) that enables Cypher queries, vocabulary support, provides auto-complete functionality, and URI resolution services. With this step, we enable universal ApiNATOMY data accessibility and interoperability with other biomedical KR systems.

### ApiNATOMY Knowledge Representation

As a KR format, ApiNATOMY has four major requirements: *authoring*, *visualization*, *querying*, and *validation*. Authoring is concerned with providing guidelines for users on how to define valid ApiNATOMY models. Visualization and querying are both critical for enabling authoring and validation.

#### Authoring

ApiNATOMY models can currently be authored in two ways. They can either be written directly in JSON that matches the input model schema, or they can be created by editing a template spreadsheet that has a well defined mapping to the JSON input model schema. We are working to accept other input formats that convert to JSON in the future.

SPARC experts usually work in a spreadsheet but can write JSON directly as well in some cases. Individual ApiNATOMY resources representing anatomical entities are mapped to community ontology identifiers during the authoring process. If an existing term cannot be found then it is added to InterLex (interlex.org). Using ontology IDs at this stage of authoring is key to achieving FAIR.

After initial syntactic and structural validation, the input model is further processed by the transformation pipeline to create resources necessary for the generation of subgraphs described in the model templates. Once the *expanded* model is ready, it is visualized in the main ApiNATOMY viewer window.

#### Visualization

Visualization of ApiNATOMY models is a crucial element of the validation process, and as a means for users to interact with the knowledge via a schematized, visual interface. Visualization provides a way for authors to rapidly check the correctness of a model that is complementary to textual competency queries. In addition, for those users of ApiNATOMY who are not authors, snapshot-style displays of connectivity (see below) are key for walking through a complex visual model.

ApiNATOMY models can be visualized using the *ApiNATOMY viewer* (Open-Physiology Resource Viewer, 2021; Open-Physiology-Viewer [2017], 2021; Kokash et al., 2022). The viewer is a client-side web application implemented using WebGL and Angular. A typical model includes authoring and publication metadata such as the ID, name, author, version, imports, etc. as well a specification of all the resources needed to describe the conduits and groups that are part of the model. The ApINATOMY viewer allows users to create new models or load existing ones from the local file system or external repositories. It is also possible to compose new models by joining existing ones. The model IR can be reviewed in the embedded code editor and exported in JSON format for reuse or in the expanded JSON-LD format for integration with other resources.

While meaningful, contextual representations of biological systems in ApiNATOMY should ideally include both connectivity and scaffold parts, each of the model types can be rendered separately by the viewer: this is useful for visual inspection and analysis of models in development. Without any explicit layout constraints (that are provided by scaffold components), a connectivity model is displayed using a force-directed graph layout. The method displays a large graph by positioning its unconstrained nodes in two-dimensional or three-dimensional space so that all edges are of required length and there are as few crossings as possible. Constrained nodes have to be positioned into a certain place, either defined explicitly in the input model or determined by their relation to other elements, e.g., it is common for graph nodes to be placed on the borders or inside of conduits.

Hence, our visualization algorithm first creates WebGL objects for all visual components, then starts a force-directed simulation, updating dependent resources on each iteration: *links* are updated to connect nodes that changed position, *lyphs* (abstracted models of conduits) are scaled, translated and rotated to align with their axes, generated chain *nodes* are placed on borders or inside of housing lyphs (i.e., lyphs that house other lyphs), and housing lyphs that bundle some chains are incentified to be positioned next to each other.

While graph drawing is generally a difficult problem, especially for large complex graphs that we typically encounter in ApiNATOMY models, the force-directed algorithm, being a physical simulation, requires no special knowledge about graph properties such as planarity, and provides a generic default layout that can be further refined for each model individually. If the initially produced layout is not very intuitive, the force-directed graph can be stimulated to rearrange the graph nodes. The ApiNATOMY viewer also provides a number of tools for graphical interaction with the model. Users can change the view point by moving the 3d view camera, zooming in and out, toggle on and off 3d scene interactive controls (interactive scaffold editing is more convenient to do on a fixed snapshot), return view camera to the initial position, enable or disable antialiasing, resize text labels, provides support for quality control via the access to logging messages and the execution of queries against external repositories, as well as to download the full generated model and resource map.

Detected structural or logical issues are reported via a specialized logging service. The status of the model can be seen via an icon on the model toolbar:

• The green circle indicates that the model is correct: no issues were detected in the imported model.
• The yellow triangle on the button indicates the presence of warnings, or non-critical errors; these issues must be revised as they are likely caused by wrong resource definition or incorrect relationships, but the overall model is unlikely to be affected.
• The red triangle indicates that there is a critical error in the model. The model may still be displayed, but nothing is guaranteed, the integration with SciGraph is likely to fail.

If the model includes instructions to import other models, an extra menu button appears. It opens a dialog with URLs of requested models, allows users to select which models to import, and displays the statuses of corresponding HTTP requests.

**FIGURE 3:**
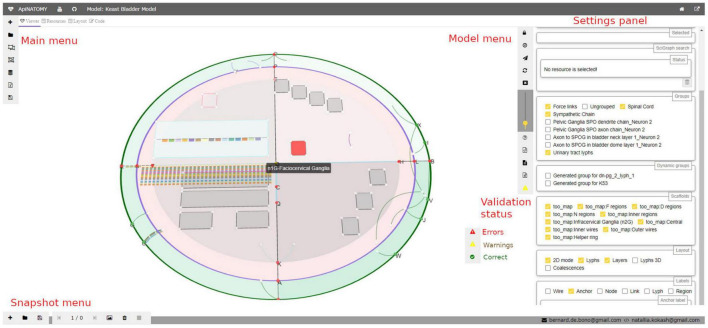
A snapshot of the *ApiNATOMY viewer* web application. In the main view, a user can see an ApiNATOMY model of the Spinal Cord, Sympathetic Chain, and Urinary Tract, placed in the context of the TOO Map. On the left hand side, there is the *main menu* for loading and composing models. On the right hand side, there is the *model menu* that provides controls for the interaction with the currently displayed model, and the *settings panel* that provides access to relevant model and visualization parameters. At the bottom of the model menu users can see model status: the demo model has *warnings.* At the bottom of the viewer, there is the snapshot *menu* to create visualization snapshots and switch between recorded model states.

The *settings panel* in the right side of the viewer (see Figure 3) provides further options for navigating the model. It displays selected properties of a highlighted visual resource, helps to find conduits by name, allows users to configure which parts of the model (groups) are visible on the screen, switch on and off various resource labels (IDs or full names of nodes, links, lyphs, etc.), and change model layout parameters.

The *scaffold models*, such as TOO map, are processed in a similar way. These models include different types of resources: *anchors*, *wires*, *regions*, and *components*. Scaffold resources help us to delimit space and position of connectivity model resources in an intuitive way, but they do not define biological tissues, i.e., conduits and process flows are not part of a scaffold model. Users can manipulate visual layout by dedicating certain space to certain subsystems (connectivity model groups). The ApiNATOMY viewer allows users to relocate anchors and resize regions by dragging wires in the graphical environment. The updated scaffold can be downloaded and saved as a new stand-alone scaffold model or included into a snapshot model.

**FIGURE 4:**
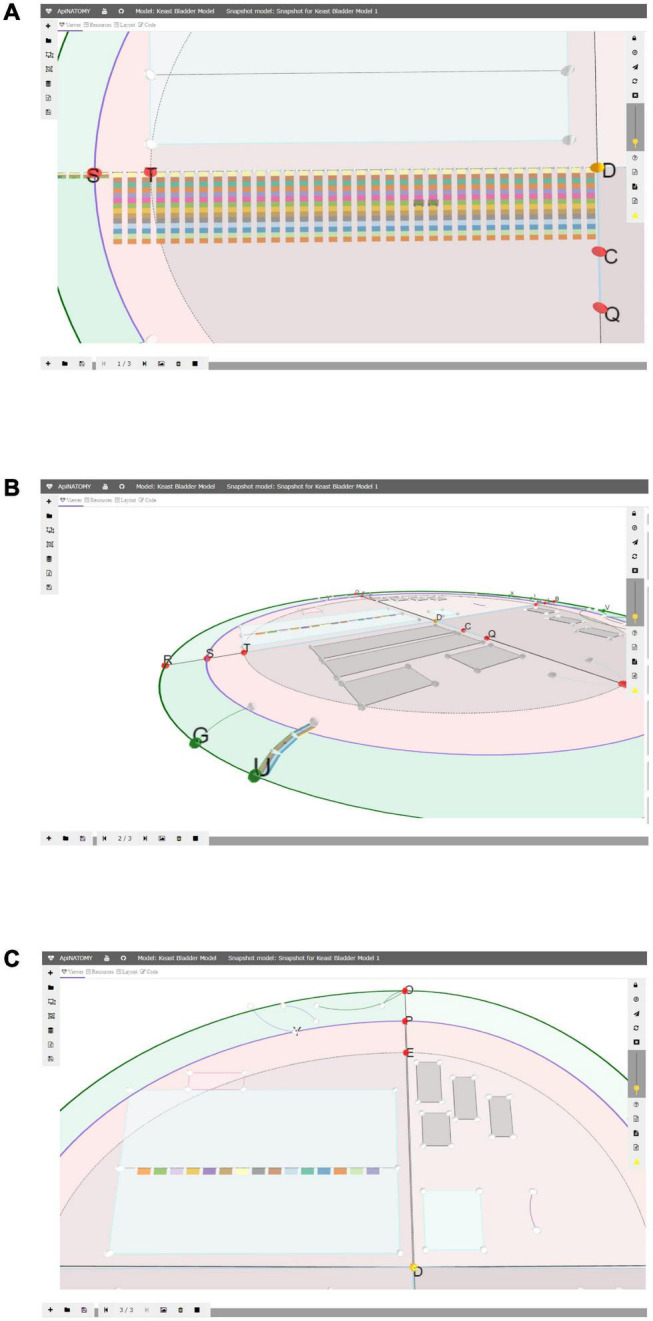
A snapshot model with three states: in panel **(A)** we emphasized the layered ApiNATOMY representation of the Spinal Cord; in panel **(B)** we show a Urinary Tract chain (U) from a different camera angle; in panel **(C)** we resized segments of the TOO Map and reshaped some scaffold regions, most notably, Infracervical Ganglia region featuring basic elements of the Sympathetic Chain.

The *snapshot model* (Figure 4) is a recording of a view camera and scaffold anchor positions. By storing and reloading this information, one can create animations, or transitions from one view into another. The viewer provides controls for creating new snapshot models, saving them in files, and loading existing models. Each model consists of one or more states, states can be added, removed, or reordered. A user can switch between them, updating the scaffold elements and camera parameters in the main view.

#### Querying

As stated in Grüninger and Fox (1995), the role of modern information systems is not only to show what is explicitly represented in the model, but deduce answers to queries based on the IR of domain knowledge. Our querying infrastructure is critical for practical applications of knowledge we accumulate, and is also used as part of the validation process to run competency queries that test the integrity and correctness of the ApiNATOMY models. As mentioned in the infrastructure overview, we make use of two KM systems to query ApiNATOMY models: RDF and property graphs. For these resources, we use Blazegraph and Neo4j, respectively. The pipeline that populates the querying infrastructure involves the following steps:

(1) An authored spreadsheet is converted into the input JSON format.
(2) The input JSON format is converted into the expanded JSON-LD model where all elements have unique local identifiers. This representation defines a graph convertible to RDF in a single step using a *JSON-LD 1.1 @context* (Context Definitions in Json-Ld 1.1, 2021) without modifying the original representation.
(3) Lightweight OWL semantics are applied to the ApiNATOMY model, and individual neuron populations are treated as *NamedIndividuals* (FullSemanticsNamedIndividuals - OWL, 2021), while external identifiers are treated as *Classes*. In principle this makes it possible to validate the structure of neuron populations defined by ApiNATOMY models against lighter neuron types from the Neuron Phenotype Ontology (NPO) (Gillespie et al., 2020). Neuron populations are sets of neurons that share defining properties that distinguish them from other similar populations. For example, there may be many populations that have their somas located in the Superior Cervical Ganglion, however, they can be differentiated by considering their projection targets, both anatomically and based on their target populations. For the parts of the NPO that are related to SPARC, the major defining properties for the populations are the locations of their somas, axons, and dendrites. The intersection between neurite type and anatomical region is usually sufficient to uniquely identify the populations in ApiNATOMY models.
(4) The JSON-LD output is then serialized as Turtle file (.ttl), and loaded into a triple store or converted into a Neo4j property graph via OWLAPI as part of SciGraph.
(5) Once an ApiNATOMY model is loaded and accessible in one of these stores it is possible to write queries to ensure that the information that we expect to be in the model can be verifiably retrieved.

#### Validation

There are five core aspects that need to be validated for each ApiNATOMY model, reflected in the following high-level competency queries:

(1) Are the groups of conduits assembled correctly? Do they have the topology that we expect?
(2) Are the conduits in the right location within the larger housing model? For example, does neuron population *X* actually terminate its axons in region *Y*?
(3) Is the branching structure of conduits correct?
(4) Are the number of distinct anatomical locations and individual elements in the model matching our expectations?
(5) Does the model match the source textbook diagram or SPARC expert’s view?

ApiNATOMY model *validation* aims to answer the above questions with the help of the following tools in our infrastructure:

(1) Visual checks matching the structure of neuron populations rendered in the *ApiNATOMY viewer* with the corresponding hand-drawn diagrams produced by experts or found in textbooks. Visual checks are also made more effective by the use of specific materials to e.g., distinguish between axons and dendrites, and are aided by the fact that conduit types Bags and Tubes have slightly different colors. This feature allows the author to quickly see whether the topology of the model is correct and whether its parts are made of the right materials.
(2) An important instrument for model validation is provided by the automatic creation of dynamic groups of closed subsystems: in a correctly assembled model, structures composed of sets of lyphs of type BAG and type TUBE usually form fluid containing boundaries. These connected subgraphs can be thought of as topologically equivalent to a lyph of type CYST (e.g., a cell). We use a *depth-first search* algorithm to find all such components, and optionally associate them with group parameters via a *seed* which is a conduit that such a dynamic group must include (e.g., a specific named neurite or soma). These *dynamic groups* are critical for being able to validate model correctness. We often refer to the discovery and creation of dynamic groups as *neurulation* since this process allows us to assemble neurons and neuron populations from model parts by constructing the subgraphs that are topologically equivalent to a cyst.
(3) Review of the outputs of competency queries written in Cypher or SPARQL. There are two main queries that we use to validate the larger scale structure. One is to list all the locations where there are neural processes that are part of a specific neuron population (soma processes), and the other is to list all the populations that pass through a given anatomical location. These checks can also be automated by independently authoring the simpler and easier to validate NPO neuron types. A worked out example of such queries is illustrated in Figure 2(ii).
(4) Checks to ensure that the number of actual groups with certain topological properties matches the expectations. We have developed a set of competency queries that can be checked automatically, and another which requires human review. In the first case we ensure that the number of groups that are topologically equivalent to a cyst (e.g., neurons and cells) and that are subclasses of neurons match the number of neuron types that we expect in the model. Other numerical error detection and correction-like queries can also be carried out for the number of neurites, etc.

We also plan to implement a fourth validation method which uses the NPO representation of neurons (which is easier to use for data entry) as a type check on the more complex structure of the ApiNATOMY models of the neuron populations.

## Methods

### Depicting Canonical Routes

This subsection has two parts:

(a) By way of example, this sub-section starts by outlining the graph construction of a canonical TOO map for mammalian anatomy. The TOO map (which stylistically revisits a historical technique in map making known as the T and O Map (2021) serves as a visual wire-frame representing principal transport thoroughfares in the body. This wireframe acts as a graphical scaffold that anchors and lays out conduits found in canonical connectivity models.
(b) The authoring of an exemplar connectivity map, in this case detailing the neuronal inputs and outputs of the superior cervical ganglion, is presented in the second part of this sub-section. Here, we also show how this connectivity model is anchored onto the TOO map.
• Part I: TOO Map exemplar: mammalian anatomy

A TOO map consists of interlinked nodal, linear or rectangular graphical elements. The mammalian TOO map visually apportions, and depicts as lines, three principal categories of extracellular material, as follows:

• in blue, a “T”-shaped depiction of CerebroSpinal Fluid (CSF) (Figure 1: blue edges connected by nodes H, N, T, C and Q),
• in red, an inner “O”-shape denotes the circulation of blood (Figure 1: red edges connected by nodes L, A, S, Y and P, with node D as reference centroid for this ellipse), and
• In green, the outer “O”-shape denotes the flow of surface materials on the surface of the body, such as digestive juices, food, chyme, chyle, feces, air, sweat, tears, mucus, urine, milk, reproductive fluids, and products of conception (Figure 1: green edges connected by nodes B, V, J, W, M, U, G, R, O, X and Z).

In addition to visually illustrating the transport materials listed above (i.e., CSF, blood, and surface materials), the mammalian TOO map graphically constrains the display of other classes of extracellular material thoroughfares to consistent locations on screen:

• Other red lines: lymphatic system, two blood portal systems; three mesothelial fluid sacs (pleural, pericardial, and peritoneal).
• Red rectangle: Tissue fluid of the spleen.
• Other blue line: Tissue fluid of the cardiac conduction system.
• Blue rectangles: Tissue fluid of skeleton, fascia, ligaments and skeletal muscle; synovial fluid of joints; endoneurial fluid of the nerves and ganglia.

The TOO map is authored through the definition and semantic annotation of its nodal, linear and rectangular elements in a spreadsheet that serves as an input form to the ApiNATOMY graphical viewer. A sample tabulation of the key entries managed by the spreadsheet for the mammalian TOO map is provided in Table 2.

**TABLE 2:** Sample definitions of elements that constitute a mammalian TOO map.

Element type	ID	Name	Definition or external term ID
Node	B	Buccal	Boundary between the oral cavity and the skin at the vermillion border
Node	Z	Junction of salivary glands with oral mucosa	Boundary between salivary ducts and the oral cavity
Node	f1S	Acinar end of salivary glands	Cul-de-sac of the salivary arborization
Node	X	Junction of esophagus, larynx, and laryngopharynx	Junction of esophagus, larynx, and laryngopharynx
Node	f1L	Alveolar end of airways	Cul-de-sac of the airway arborization
Node	O	Junction of ampulla of Vater with duodenum	Junction of ampulla of Vater with duodenum
Line	w-J-f4P	Conjunctiva	UBERON:0001811
Line	w-J-V	Nasal Skin	FMA:24763
Line	w-V-f4N	External Nasal Mucosa	FMA:59685
Line	w-f4N-f4P	Nasolacrimal Duct	UBERON:0002392
Line	w-f4P-f4L	Lacrimal Tract	UBERON:0001817
Line	w-f4N-f4E	Internal Nasal Mucosa	FMA:59661
Rectangle	n3U	Upper Limb Skeleton	UBERON:0001440
Rectangle	n4c	Cranial Skeleton	UBERON:0003128
Rectangle	n4V	Vestibulocochlear organ	UBERON:0002227
Rectangle	n4R	Eye	UBERON:0000966
Rectangle	n4O	Olfactory bulb	UBERON:0002264
Rectangle	n4C1N	Cranial Nerve 1 (CN1)	UBERON:0001579

**FIGURE 5:**
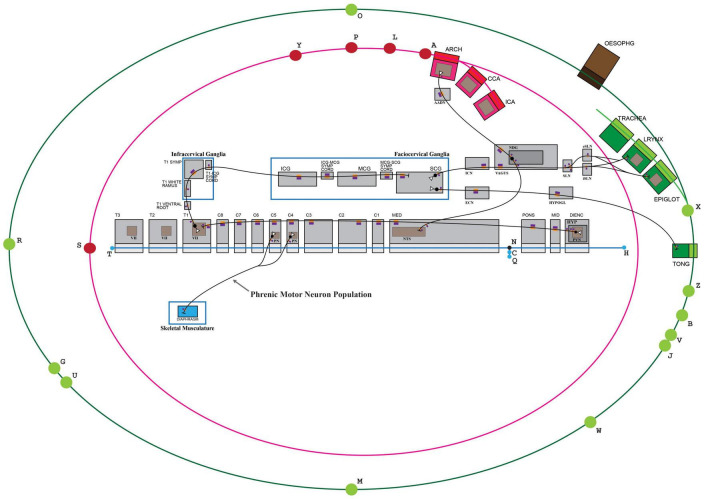
TOO map providing a visual context to an ApiNATOMY connectivity model. Some elements of the TOO map that are not required to anchor the connectivity model have been concealed for ease of viewing. The blue edge defined by nodes (H,T), representing the flow of CSF, serves to organize conduits representing contiguous structures in the Central Nervous System. KEY: Black lines represent the flow of neuronal cytosol; Black circles represent neuronal somata; Open white triangles represent the dendritic terminus of a neuron; Purple-and-orange rectangles represent segments on neurons (orange: innermost, cytosolic layer; purple: plasma membrane); T1: first thoracic; T1 SYMP: sympathetic ganglion at the level of T1; ICG: inferior cervical ganglion; MCG: middle cervical ganglion; SCG: superior cervical ganglion; ICN: internal carotid nerve; ECN: external carotid nerve; HYPOGL: hypoglossal nerve; NDG: nodose ganglion; SLN: superior laryngeal nerve; iSLN: internal SLN; eSLN: external SLN; LRYNX: larynx; EPIGLOT: epiglottis; TONG: tongue; OESOPHG: esophagus; NTS: nucleus tractus solitarius; ARCH: arch of the aorta; CCA: common carotid artery; ICA: internal carotid artery; AADN: aortic arch depressor nerve.

• Part II: Connectivity model exemplar: Superior Cervical Ganglion (SCG) model (Figure 5)

In ApiNATOMY, the overall approach to schematizing neural connectivity is to model the flow of neuronal cytosol as processual edges that are conveyed by layered conduits representing segments of a neuron. In such a scenario, a neuronal segment is defined via a conduit template with three layers:

• an innermost layer (in which the processual edge representing cytosolic flow is embedded) annotated with the GO Cell Component term for “Cytosol”,
• a middle layer annotated with the GO Cell Component term for “Plasma membrane”, and
• an outer layer annotated with the GO Cell Component term for “Extracellular Fluid”.

The linear assembly of template-derived conduits representing a neuron requires the following minimal features:

• at least one conduit of type Tube or Bag representing the soma segment (i.e., nuclear region) of a neuron;
• at least one conduit of type Bag representing the axonal terminus of a neuron;
• at least one conduit of type Bag representing the dendritic terminus of a neuron.

ApiNATOMY’s multi-scale modeling, shown in Figure 5, of nested conduits is exemplified here in the assembly of SCG-related structures that belong to the peripheral (PNS), autonomic (ANS), and central nervous systems (CNS). The stretch of CNS from the diencephalon to the third thoracic spinal level, for instance, is created from the linear assembly of layered conduits for CSF, generated from the same ApiNATOMY conduit template. This template defines an archetypal CNS conduit of type Tube that consists of:

• an innermost layer constituted from material annotated with the term “Cerebrospinal fluid”^2^ () and
• an outer wall constituted from material annotated with the term “Parenchyma of central nervous system.”^3^

The creation of these CSF-conveying CNS conduits, described above, is followed by the embedding of unlayered conduits within their walls to represent gray-matter nuclei (such as the nucleus tractus solitarius, NTS, in Figure 5) that host conduit representations of somata and axonal or dendritic termini: the graphical nesting of a conduit within another denotes the relationship of partonomy.

Automation in the ApiNATOMY authoring workflow enables the instantiation of linear assemblies of axons or dendrites to create a contiguous set of neuron segments (via the same neuron conduit template) simply by referring to an ordered list of housing conduits in which these neuronal segments will sequentially be embedded. For instance, the axon that originates from the paraventricular nucleus (labeled PVN in Figure 5) of the hypothalamus (HYP in Figure 5) in the diencephalon (Dienc in Figure 5), and terminates in Rexed lamina VII of thoracic spinal segment T1, is constructed via the execution of an automated procedure that generates sixteen connected processual edges representing cytosolic flow, respectively conveyed by distinct instances of neuron segments that are embedded in an ordered list of housing conduits (as follows, the outer layer of: PVN, HYP, DIENC, MID, PONS, MED, C1, C2, C3, C4, C5, C6, C7, C8, T1, VII of T1). An analogous procedure is followed to thread neuronal processes along ganglia and nerves (e.g., vagus nerve or T1 white ramus, shown in Figure 5) which are featured as unlayered conduits that convey their own endoneurial fluid.

As discussed in section 2, given the approximate nature of experimentally-determined neural route topologies, it is also necessary to consider the following constraints when modeling canonical neuronal connectivity in ApiNATOMY:

• Tracing studies generate data about a population of biochemically- and electrophysiologically-similar neurons such that, in practice, neuronal representation in ApiNATOMY stands-in for a population of a neurons of the same expertly-determined class;
• Neurons are highly arborized structures. It may therefore be difficult for experts to tease out the route of an individual neuron out of a population-level dataset that is acquired by tracing studies. Modeling the route of a population of neurons, therefore, must cover an aggregate of pathways for a number of neurons, such that a model for a single neuronal class may encompass somata in distinct anatomical locations (see in Figure 5, for instance, the model for the neuronal population representing phrenic motor neurons with somata at both C4 and C5 spinal levels);
• Independently of the number of somata, axons and dendrites that a model of a neuronal class may entail, the conduit assembly for such a model must generate a maximally connected set of conduits in the Tube or Bag configuration such that the resulting structure presents no open-ended tubes. As any representation of a cell in ApiNATOMY, therefore, the overall topology of a single neuronal class model must be that of a Cyst. (*Neurulation*, or the automated checking for an overall Cyst configuration of neuronal class models is a key validation step in the ApiNATOMY workflow, as discussed in section 2 above).

To visually assist with the collaborative and iterative authoring process, connectivity models are graphically anchored to elements on the TOO map. In this case, the display of the canonical SCG connectivity model depicted in Figure 5 is constrained as follows:

• the linear assembly of three layered conduits representing large arteries—aortic arch (ARCH), common carotid artery (CCA) and internal carotid artery (ICA)—is anchored to the nodal element “A” that represents the boundary between the left ventricle and the aorta at the aortic valve;
• the linear assembly of three layered conduits representing CNS structures — diencephalon (Dienc), midbrain (MID) and pons (PONS)—is strung out along the linear element “H-N” that represents cranial CSF thoroughfares starting at the fourth ventricle;
• the layout of unlayered conduits representing the inferior (ICG), middle (MCG), and superior (SCG) cervical ganglia, as well as their two intervening neural cords, is bound by a rectangular element labeled “Faciocervical ganglia” which contains conduits representing ganglia of the head and neck.

### Prototyping the Depiction of Pathologies as Realizable Routes

In de Bono et al. (2016a), we considered the classification of pathological mechanisms from the perspective of the “*creation, destruction and alteration of routes*.” In that work, we observed that mechanisms in normal (or canonical) physiology, pathophysiology, as well as therapeutic intervention, can be classified in terms of transfers between compartments in the body.

This classification of pathological mechanisms is progressed further in this subsection by:

• defining three core categories (labeled A.C) of pathological mechanism in terms of distinct transfer characteristics, as well as
• identifying specific examples of these three core categories in terms of an altered pairwise transfer between two compartmental thoroughfares of the TOO map.

In particular, Supplementary Table 1 presents an all-vs-all square symmetrical matrix of pairs of TOO map compartments, such that each pair is linked to a known pathological mechanism of exchange transfer between the two compartments. This table is available as a spreadsheet in the Supplementary Material section.

Nine examples of specific pathological mechanisms from Supplementary Table 1 are elaborated below, in terms of an A.C mechanism category to which they have been classified, as a means to outline the rationale to our adopted approach. In this approach, a mechanism is considered pathological if the transfer between compartmental thoroughfares:

• [Category A] occurs at a flow rate/pressure/resistance that is outside the range measured in the normal population, for example:
  • the occlusion of a canonical connection [e.g., choking due to blockage between the laryngopharynx in the upper respiratory tract and the trachea in the lower respiratory tract (Airway, Nasal Pharyngeal)];
  • the alteration of permeability at the exchange interface [e.g. the deposition of amyloid between blood vessels in the glomerulus and epithelial tubing of the nephron’s Bowman’s capsule alters the permeability of the capillary-to-nephron interface leading to nephrotic syndrome due to loss of plasma proteins to the urine (Urinary, Blood)];
  • the alteration of hydrostatic, osmotic or oncotic pressure to maintain canonical rates of flow [e.g., when the blood arterial system is in a state of chronic hypertension, normal cardiac output can only be achieved at the cost of an increased driving pressure against a higher-than-normal total peripheral resistance (Blood, Blood)];
• [Category B] transmits, over a canonical route, material that is not canonical, for example:
  • the transfer of microorganisms [e.g., a bacterial infection of the CSF compartment, such as meningitis, leading to the transfer of bacteria into the blood compartment causing septicemia (CSF, Blood)];
  • tumor metastasis [e.g., prostate cancer, in the reproductive system, spreading through the prostatic capsule along tracks of endoneurial fluid of the innervating hypogastric plexus (Reproductive, Endoneurial)];
  • infiltration of leukocytes in autoimmunity [e.g. in multiple sclerosis, the altered permeability in the blood-brain barrier causes entry of T-cells into the CSF, a key event in the destruction of myelin (CSF, Blood)];
• [Category C] occurs along a non-canonical route, for example:
  • the failure of conduit integrity leading to leakage [e.g., in hemorrhagic stroke, arterial wall integrity is eroded due to atherosclerosis (Blood, CSF)];
  • the abnormal development of conduits leading to the creation of non-canonical routes [e.g., a patent ductus arteriosus after birth allows blood under high pressure from the aorta to enter the pulmonary arterial tree (Blood, Blood)];
  • immune-mediated cell-destruction mechanisms that create non-canonical conduits within the cell’s plasma membrane that short-circuit the regulation of flow between cytosol and extracellular fluid (e.g., the Perforin complex is released from cytotoxic T-cells to create pores on the membrane of the cell under attack, allowing for the entry of apoptosis-inducing granzymes).

In prototyping this representational approach, two key operational principles emerge when classifying pathological mechanisms in terms of altered transfer properties, namely that:

• the three (A.C) categories of altered transfer may contribute to varying degrees to the mechanism of a complex pathology at different points along its course (e.g., in the case of malignancy, the category-C creation of non-canonical routes may lead to the transfer of category-B non-canonical material, such as tumor cells or microorganisms; in multiple sclerosis: the blood-brain barrier breaks down [Category C] allowing T-cells to move into the CSF of the affected region [Category B], causing cytotoxic T-cell attack on oligodendrocytes [Category C], which leads to the slowing of action potential flow rates due to ensuing demyelination [Category A]);
• the modeling of pathological mechanisms in terms of altered transfer is dependent on the granularity of compartment representation (e.g., in this work, the set of compartmental pairs selected for mechanism classification was chosen from the compartmental thoroughfares shown in the TOO map). A higher resolution map of pathology, defined in terms of category A.C mechanisms, may be achievable if multi-scale connectivity models of these thoroughfares were available).

## Results

Within the SPARC effort, the methods described above result in SCKAN, the SPARC knowledge base of the autonomic nervous system (Gillespie, 2021b). SCKAN is an integrated graph database composed of three parts:

• a metadata graph that documents and organizes the experimental datasets generated by the SPARC consortium;
• models of connectivity and their scaffolds that link to the above metadata graph via ontology-based semantic annotation (drawn from the reference ontology below) on the basis on the JSON schema published in Gillespie and Kokash (2021);
• a reference ontology, which is a combination of the NIF-Ontology (Imam et al., 2012; “Neuroscience Information Framework (NIF) Standard Ontology - Summary | NCBO BioPortal, 2021”; “NIF | NIF Vocabularies, 2021”) and community ontologies (e.g., Rosse and Mejino, 2003; Bard et al., 2005; Hastings et al., 2013; Haendel et al., 2014; Groza et al., 2015).

Table 3 shows SCKAN statistics about a sample of its connectivity models.

**TABLE 3:** Content statistics for a sample of ApiNATOMY connectivity models in SCKAN.

Graph URL	Input Lyphs	Total Lyphs	External References	Publications	Neuron Populations	Model Label
https://apinatomy.org/uris/models/keast-bladder/ids/keast-bladder	139	2561	175	25	20	Bladder
https://apinatomy.org/uris/models/bolser-lewis/ids/bolser-lewis	107	499	168	238	29	Superior Cervical Ganglion
https://apinatomy.org/uris/models/bronchomotor/ids/bronchomotor	143	554	135	6	6	Bronchomotor control
https://apinatomy.org/uris/models/ard-arm-cardiac/ids/ard-arm-cardiac	136	1976	146	1	17	Heart
https://apinatomy.org/uris/models/sawg-distal-colon/ids/sawg-distal-colon	85	960	118	4	17	Distal colon
https://apinatomy.org/uris/models/sawg-stomach/ids/sawg-stomach	38	751	75	8	14	Stomach

*The columns are defined as follows: Graph URL: URL for the graph of the ApiNATOMY connectivity model; Input Lyphs: number of lyph conduit objects explicitly defined by the authoring modelers; Total Lyphs: number of lyphs conduit objects in the expanded model; External References: number of external resources (e.g., ontology terms) linked to the ApiNATOMY resources; Publications: number of publications linked to the model; Neuron Populations: number of neuron populations; Model Label: name of the connectivity model.*

SCKAN is distributed as two Docker images, one with the software needed to run queries, the other containing only the loaded databases. Instructions for getting SCKAN up and running on Linux, Mac OS and Windows systems are available here (Gillespie, 2021c). We provide authors detailed instructions for the SCKAN release process. For the SCKAN data release, the following four steps are needed:

• ApiNATOMY model RDF export and deployment (Gillespie, 2021a).
• SciGraph deployment (Gillespie, 2021d).
• Knowledge base release (Gillespie, 2021f).
• Blazegraph deployment (Gillespie, 2021b).
• All the above are archived as part of (Gillespie et al., 2021a).

To create an accessible version of the knowledge base that can be queried, we convert and enrich the SPARC database by loading it both into a Blazegraph triplestore as well as a Neo4j property graph. These two representations serve complementary use cases:

• the triplestore is useful for executing basic competency queries over the dataset releases: however, no APIs are available that are straightforward for developers to consume;
• the SciGraph property graph provides a developer-friendly REST API that is much easier to use in production systems.

ApiNATOMY client-side applications run queries using the SciGraph REST API. Technical Cypher formulations of such queries are available here (Gillespie, 2021g). These formulations include search queries used for validation of ApiNATOMY models under development, for instance:

• *Return the paths to somas from an anatomical region*.
• *List all the somas for a given model.*
• *List all the neuronal processes for a given starting point*.
• *List all the neuronal processes for given somas*.

The documentation pages providing more detailed examples of queries in relation to the ApiNATOMY models are available here (Gillespie, 2021e). For example, in return to a query “*Find all of the anatomical regions that neuron population #5 travels to in the Bladder model”*, the SCKAN retrieves knowledge about the neural circuits (for neuron population #5 in the Bladder model) innervating the urinary bladder and urethra. The neurons in this neuronal circuit, neuron population #5, refers to a population of parasympathetic preganglionic neurons with somas in L6 and S1 spinal cord segments, and with axon terminals in the pelvic ganglia.

Apart from the predominantly neural use case that motivates the SPARC effort, the broader discovery requirements elicited from the CFDE effort (as outlined in section 2.1.1) seek a generalizable approach to classify and reason over any mechanistic disease knowledge across scales. The preliminary results described in Supplementary Table 1 provide a breadth-first low-resolution map of pathology knowledge, applied to any modality of biological connectivity, in terms of three types of miscommunication (categories A.C) between ApiNATOMY conduits. While the prototyping of this knowledge representation is in its early stages, in section 5 we discuss our next steps in extending the ApiNATOMY schema for the associated workflow to sustainably encompass pathology knowledge in SCKAN.

## Discussion

The ApiNATOMY method seeks to progress on the representation of biological pathways which, in the field of systems biology has been primarily focused on databases depicting pathways of biochemical transformation from one molecular species to another (e.g., Orchard et al., 2014; de Bono et al., 2016b; Baltoumas et al., 2021; Kanehisa et al., 2021). Specifically, ApiNATOMY manages knowledge about anatomical pathways that are represented in the form of conduits for multiscale transport (i.e., pathways of linked conduit compartments in which, for instance, molecular species reside).

The SPARC objective to develop electroceuticals provides a key driving use case for the further evolution of the ApiNATOMY method. In particular, the requirement to assist in the planning of interventions on (and recordings from) a target region on the nervous system creates the need to access data about previous experiments carried out on neurons that pass through this target region. For instance, given a procedure planned on the Middle Cervical Ganglion (MCG in Figure 4), a researcher has the following questions: (a) *if I impale a tract or nerve with an electrode, from what neurons am I recording?* (b) *if I sever a tract or nerve, what neurons am I interrupting?* To that end, the following queries can now be directly addressed by SCKAN:

(A) what neurons are coursing through the MCG target region? (i.e., in terms of the ApiNATOMY KR, what groups of lyphs representing neurons have at least one lyph in the MCG housing lyph)?
(B) in what locations do the conduits found in step A, that belong to neurons that pass through the MCG, reside? (i.e., find the housing lyphs that contain dendritic segments, somata, and axonal segments of neurons identified in step A above).
(C) retrieve experimental metadata that is associated with the locations identified in step B above.

In effect, ApiNATOMY addresses the general requirement, originally articulated in de Bono et al. (2016a) to “*take into account correlations between biomedical measurements. In some cases, it is important to understand how the route linking the locations of measurement contributes to the correlation.*” In some cases, such a route accounts for correlations that typify a canonical physiological process, in other cases a pathological one.

The ApiNATOMY infrastructure enables the FAIR representation of connectivity. Considerable effort has been focused on eliciting, unifying and formalizing ApiNATOMY data representation in order to provide a generic multi-scale modeling toolset. Here we show that the resulting workflow can be productively leveraged to generate detailed connectivity models. The evolution of the ApiNATOMY method is not complete given that: (i) the authoring of ApiNATOMY models still remains a labor-intensive task and (ii) the scalable inclusion of disease knowledge is not yet supported. To address these obstacles, the collection of requirements from authors and modeling experts has elicited a development roadmap, which prioritizes on the following six areas for further work to:

• design guidelines and reusable patterns that foster the efficient production of correct and accurate ApiNATOMY models;
• improve the visual representation and map interaction to reduce the time authors spend on validating their models;
• increase the anatomical coverage of the connectivity map by accelerating connectivity model production through the expert-supervised application of natural language processing methods. This approach is expected to accelerate the harvesting of statements that provide peer-reviewed evidence, and thereby create a whole-body reference connectivity model that:
  • can be leveraged to organize, classify and search metadata drawn from heterogeneous sources (i.e., relate, on the basis of connectivity, metadata for resources beyond SPARC, such as those provided by the CFDE effort);
  • provides increasing levels of connectivity model resolution in support of a finer-grained description of pathological mechanisms in terms of altered connectivity;
• extend the ApiNATOMY schema to encompass knowledge about the three categories of disease mechanism as follows:
  • [Category A] knowledge representation for properties of flow will leverage the biophysical modeling results reported in de Bono et al. (2018) where an implementation of the Bond Graph formalism was applied to ApiNATOMY. In that work, ApiNATOMY edges and lyphs were adapted to link to Bond Graph representations of resistive, inertial and elastance features of flow. From the perspective of Category A knowledge management, ApiNATOMY conduits correspond to the field of 0-nodes (as defined in the Bond Graph formalism), and are semantically equivalent to a well-mixed, equipotential fields tracked by a μ variable (representing the Bond Graph concept of a *driving potential*) associated with an 0-node.
  • [Category B] this category of pathology will require a specific schema adjustment to enable the categorization of flow materials as either canonical or non-canonical with reference to a particular material-conveying edge in ApiNATOMY.
  • [Category C] the ongoing development of generalizable solutions in ApiNATOMY to model connectivity variants (e.g., solutions to represent canonical routes for orthologous neural pathways that are found in different species) is anticipated to be relevant to depict aberrant (i.e., non-canonical) routes for Category C pathologies. Associating pathology knowledge with the models of connectivity variants will require due consideration to the nature of the material such a route conveys.

## Data Availability Statement

The original contributions presented in the study are included in the article/Supplementary Material, further inquiries can be directed to the corresponding author.

## Author Contributions

BB was main author for sections Introduction, Methods, and Discussion. TG and NK share equal authorship for sections ApiNATOMY Infrastructure and Results. TG wrote the queries. MCSZ created and curated the ApiNATOMY models. MM reviewed and curated the models. JG helped to develop the ApiNATOMY infrastructure component. All authors reviewed the manuscript.

## Conflict of Interest

The authors declare that the research was conducted in the absence of any commercial or financial relationships that could be construed as a potential conflict of interest.

## Publisher’s Note

All claims expressed in this article are solely those of the authors and do not necessarily represent those of their affiliated organizations, or those of the publisher, the editors and the reviewers. Any product that may be evaluated in this article, or claim that may be made by its manufacturer, is not guaranteed or endorsed by the publisher.

## Footnotes

1 An automated version of this pipeline is implemented in https://github.com/SciCrunch/sparc-curation/blob/master/docs/apinatomy.org which uses https://github.com/open-physiology/open-physiology-viewer/blob/master/wrapper/bin/converter.js behind the scenes.

2
http://purl.obolibrary.org/obo/UBERON_0001359

3
http://purl.obolibrary.org/obo/UBERON_0005158

## Supplementary Material

Supplementary Table 1 Pairwise-transfer pathology matrix. See spreadsheet to access the full details of this matrix.
